# Computational wear of knee implant polyethylene insert surface under continuous dynamic loading and posterior tibial slope variation based on cadaver experiments with comparative verification

**DOI:** 10.1186/s12891-022-05828-2

**Published:** 2022-09-19

**Authors:** Alaettin Ozer

**Affiliations:** Department of Mechanical Engineering, Yozgat Bozok University, Yozgat, Turkey

**Keywords:** Total knee replacement, Finite element model, Wear, Posterior Tibial slope, Continous dynamic loading

## Abstract

**Background:**

The effect of posterior tibial slope on the maximum contact pressure and wear volume of polyethylene (PE) insert were not given special attention. The effects of flexion angle, Anterior-Posterior (AP) Translation, and Tibial slope on the max contact pressure and wear of PE insert of TKR were investigated under loadings which were obtained in cadaver experiments by using Archard’s wear law. This study uses not only loads obtained from cadaver experiments but also dynamic flexion starting from 0 to 90 degrees.

**Method:**

Wear on knee implant PE insert was investigated using a 2.5 size 3 dimensional (3D) cruciate sacrificing total knee replacement model and Finite Element Method (FEM) under loadings and AP Translation data ranging from 0 to 90 flexion angles validated by cadaver experiments. Two types of analyses were done to measure the wear effect on knee implant PE insert. The first set of analyses included the flexion angles dynamically changing with the knee rotating from 0 to 90 angles according to the femur axis and the transient analyses for loadings changing with a certain angle and duration.

**Results:**

It is seen that the contact pressure on the PE insert decreases as the cycle increases for both Flexion and Flexion+AP Translation. It is clear that as the cycle increases, the wear obtained for both cases increases. The loadings acting on the PE insert cannot create sufficient pressure due to the AP Translation effect at low speeds and have an effect to reduce the wear, while the effect increases with the wear as the cycle increases, and the AP Translation now contributes to the wear at high speeds. It is seen that as the posterior tibial slope angle increases, the maximum contact pressure values slightly decrease for the same cycle.

**Conclusions:**

This study indicated that AP Translation, which changes direction during flexion, had a significant effect on both contact pressure and wear. Unlike previous similar studies, it was seen that the amount of wear continues to increase as the cycle increases. This situation strengthens the argument that loading and AP Translation values that change with flexion shape the wear effects on PE Insert.

## Introduction

Wear is the removal, surface damage, or displacement of material from one or both solid surfaces when it is subjected to contact and relative motion with another body. Operating conditions affect interface wear. The wear of components is often a significant determinant of the product’s service life.

Modeling of wear in a computational framework has been previously made effort in order to estimate the amount of PE wear that occurs at the articular surface in total knee replacements (TKR). Most of the wear studies [1–7] have been founded on Archard’s law of wear [8] by using a wear factor derived using experiments.

Osteolysis induced by particles is one of the most important reasons that limit the endurance of TKR. It is developed by the wear of bearing components made of ultra-high molecular weight polyethylene (UHMWPE) [9]. Wear can occur on the proximal surface of modular designs [9], as well as the distal surface [10–17], the surface of patella resurfacing components [18], and the post of posterior stabilized designs [9]. Wear particles can trigger an immunological response, which can set off a chain reaction of negative tissue reactions that leads to osteolysis and implant loosening [19]..

The purpose of the wear experiments of TKR is to investigate surface bearing and prothesis. Wear simulators simulate in vivo circumstances, enabling for performance evaluation of innovative designs prior to large-scale production and implantation. Researchers, manufacturers, and industry professionals can use simulator wear testing to evaluate the wear performance of their prosthesis design and bearing materials under physiological conditions. This testing can help to refine and improve their designs prior to large-scale manufacturing and implantation, in addition to meeting regulatory criteria.

Models predict wear have been earning interest within the research community as they are quicker and cheaper than experimental tests and can be easily used to study the effect of different working conditions, which could hardly be reproduced experimentally. The authors have developed some analytical models to predict wear in hip and shoulder implants. However, since they do not take into account the update of the geometry as wear evolves, they can provide useful indications for wear effects only in the short term.

Such limitations can be solved by Finite Element wear models, which are preferred for analytical solutions for simulating wear in long tests and also in cases of complex geometries. However, one of their main limitations is the computational cost. They need repetitive nonlinear contact analysis. But these limitations can be overcome by using realistic simplifications.

To anticipate pressure and wear distribution on the insert surface, Zhang et al. [20] employed a patient-specific lower extremity musculoskeletal multibody dynamics model using FEM.

Kang et al. [21] evaluated the biomechanical impact of various tibial insert materials on knee joints: UHMWPE, poly-ether-ether-ketone (PEEK), and carbon-fiber-reinforced PEEK.

Mell et al. [22] developed a computational methodology for modeling TKR wear using finite element analysis, and studied the effect of femoral center of rotation location on TKR PE wear during standardized displacement controlled testing.

Kawanabe et al. [23] created a simulator for complete knee replacements and investigated the effects of tibial AP Translation and internal-external (IE) rotation on the wear of polyethylene tibial implants. They found that IE rotation, tibial AP Translation, and rolling contributed to the higher wear rate under four types of experiments that have different loadings, suggesting that the tibial UHMWPE suffered more damage as a result of the IE.

On an in vitro knee simulator, Johnson et al. [24] evaluated the relative relevance of tibial IE rotation and femoral AP Translation and determined wear rates for the IB-II knee prosthesis for a complete normal speed walking gait cycle. They proposed that the wear rate of UHMWPE is affected by various factors such as the amount of multidirectional shear motion and the ratio of rolling/sliding contact kinematics, as well as the applied load.

The link between the posterior tibial slope (0, 7, 10 degrees), the contact force, and stresses on the medial and lateral ligaments during knee flexion following posterior-stabilized TKA was studied using FEM by Lee H.Y. et al. [25].

Using 3D finite element modeling, Shen et al. [26] studied the effect of posterior tibial slope on contact stresses in the polyethylene component of total knee prosthesis. The wear behavior of four distinct posterior tibial slopes was examined to determine the best posterior slope.

Using finite element modeling, Koh et al. [27] investigated the impact of the posterior tibial slope in mobile-bearing unicompartmental knee arthroplasty (UKA). They discovered that as the posterior tibial slope increased, contact stress increased, increasing the strain exerted on the ACL.

The effect of posterior tibial slope on the maximum contact pressure and wear volume of PE insert were not given special attention. Thus, in this study, the effects of flexion angle, AP Translation, and Tibial slope on the max contact pressure and wear of PE insert of TKR were investigated under loadings which were obtained in cadaver experiments by using Archard’s wear law. This study uses not only loads obtained from cadaver experiments but also dynamic flexion starting from 0 to 90 degrees.

## Materials and methods

### Wear model

The Archard wear model is a widely used sliding wear model that produces reasonable results when used with FEM to simulate wear. According to Archard’s original model, the contact pressure and sliding velocity at the contact surface are proportional to the rate of volume loss due to wear. The program implements a generalized version of this model that allows correct law dependence on contact pressure and velocity.

Wear is supposed to occur in the surface’s inward normal direction, which is the opposite of the contact normal direction. As a result, in Ansys, the rate of wear at a contact node is given by

$$W=\frac{K}{H}{P}^m{V}^n$$

Where, K is the wear coefficient, H is the material hardness, P is the contact pressure, V is the relative sliding velocity, m is the pressure exponent and n is the velocity exponent.

### Finite element model, analyses, materials, loading

Probably the most important part of a knee implant is the PE Insert which is made up of durable polyethylene material and which functions as meniscus in the knee. While the other pieces are metal parts with generally high solidity, the PE insert is expected to have a structure that is relatively softer, more resistant to wear, and capable of absorbing beats. For this reason, it is manufactured from UHMWPE.

**Fig. 1:**
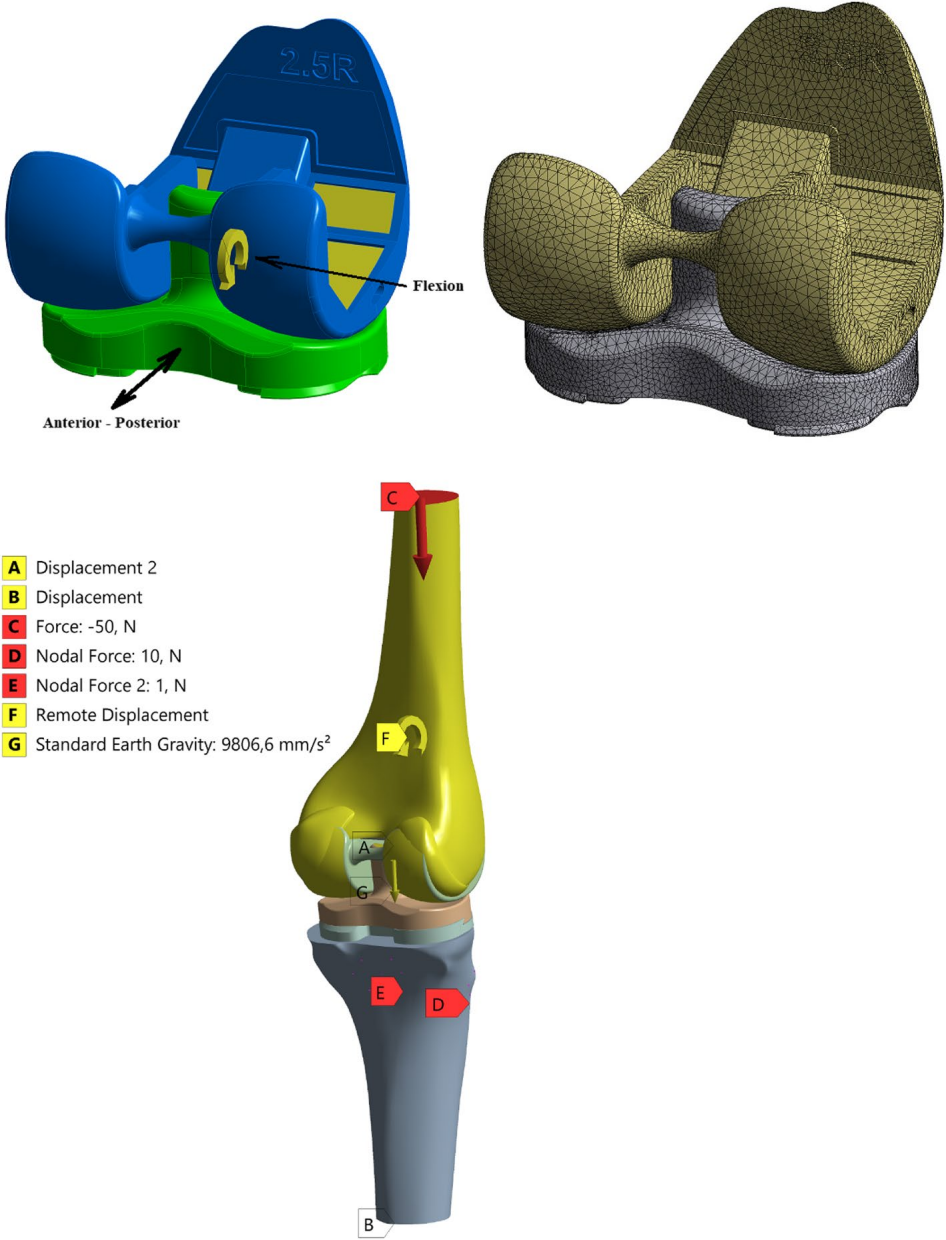
3D knee implant model (cruciate sacrificing total knee replacement) (upper left), Finite Element Model (upper right) and loading and boundary conditions on model (lower)

In this study, a 2.5 size 3D knee model obtained from Mikron Makine (Yenimahalle/Ankara/Turkey) was used (Fig. 1). Using this solid model, the Finite Element Structural Model was set up by using tetrahedral higher order solid elements. The smallest element size was 1.5 mm after mesh optimization. One hundred forty-eight thousand eight hundred five elements and two hundred twenty-three thousand sixty-nine nodes were used for the mesh (Fig. 1). Models for analyses were obtained by combining this 3D knee model with solid femur and tibia solid models by means of SpaceClaim Software. In the model matched with femur and tibia, the femur component-femur and tibia component-tibia connection places were considered as completely bonded as in real states. To simulate this, the upper end of the femur component and lower end of the PE insert were fixed. To keep femur comp on the insert while flexion, standard earth gravity was applied. The insert was fixed not to move vertically at the lower end but horizontally to model AP translation and not to move in all directions at the upper end with displacement boundary conditions (Fig. 1). Due to its continuous and dynamic nature, the contact area between femur component and PE insert was considered as frictional although it changed according to flexion angles. The friction coefficient was taken as 0.04 in line with the previous literature [28].

The following substances and values were used for the model adopted within this study. Regarding the bone properties for Femur and Tibia, the Elasticity Modulus (E) was 16.8 GPa and the Poisson’s Ratio (υ) was 0.47. Cobalt-Chromium alloy was used for the femur component (E = 195 GPa, υ = 0.3). UHMWPE was used for PE Insert with E = 685 MPa, υ = 0.47. Titanium alloy (Ti6Al4V) was utilized for Tibia component (E = 110 GPa, υ = 0.3) [28].

Two types of analyses were done to measure the wear effect on knee implant PE insert. The first set of analyses included the flexion angles dynamically changing with the knee rotating from 0 to 90 angles according to the femur axis and the transient analyses for loadings changing with a certain angle and duration. In these analyses, the PE insert was located in such a way to form angles with the femur axis by 0, 3, 5, and 7 degrees, which is named as Posterior Tibial Slope. The stable and dynamic loadings used in these analyses were ones obtained from the literature and the cadaver experiments conducted to identify and prove the loadings exerted on the knee in varying states [29]. These loadings are consisted of stable loadings of 50 N on the femur and 10 N on the hamstring and the quadriceps actuator force linearly increased and reached a force of 600 N at a 90 degrees flexion. The second set of analyses consisted of analyses of wear under the influence of AP Translation changing during flexion in addition to the flexion in the first analyses. AP translation was applied to the PE insert as one-directional displacement. The AP translation values were also adopted from the related literature [30].

In this study, wear coefficient for contacting surfaces were chosen and used as independent of contact pressure and obtained from a multi-directional pin on plate study [31]. Especially, to obtain material removal on PE insert, asymmetric contact is used.

The wear coefficient K can be scaled to simplify modeling such that the translation is not explicitly modeled, but its effect is included in the computation of wear. This significantly reduces the simulation time and effort. More specifically, if a linear dependence of wear rate on the sliding velocity is assumed, the wear coefficient K can be scaled by the sliding velocity. So, this results in the wear rate being linearly dependent upon the sliding velocity without explicitly modeling the sliding. This property is also used in this study to reduce computation time. K was scaled such that only distance taken during flexion 0 to 90 degree by femur component contacting with insert until it reaches for example 30 m cycle was scaled. This way reduce time and it is general in wear modeling. AP translation was not scaled with K. It is another boundary condition and modeled as separately.

Ansys Workbench 2020 R2 software was used to set up the Finite Element Models and carry out subsequent data analyses in this study.

## Results

### Flexion and Flexion + AP Translation

**Fig. 2:**
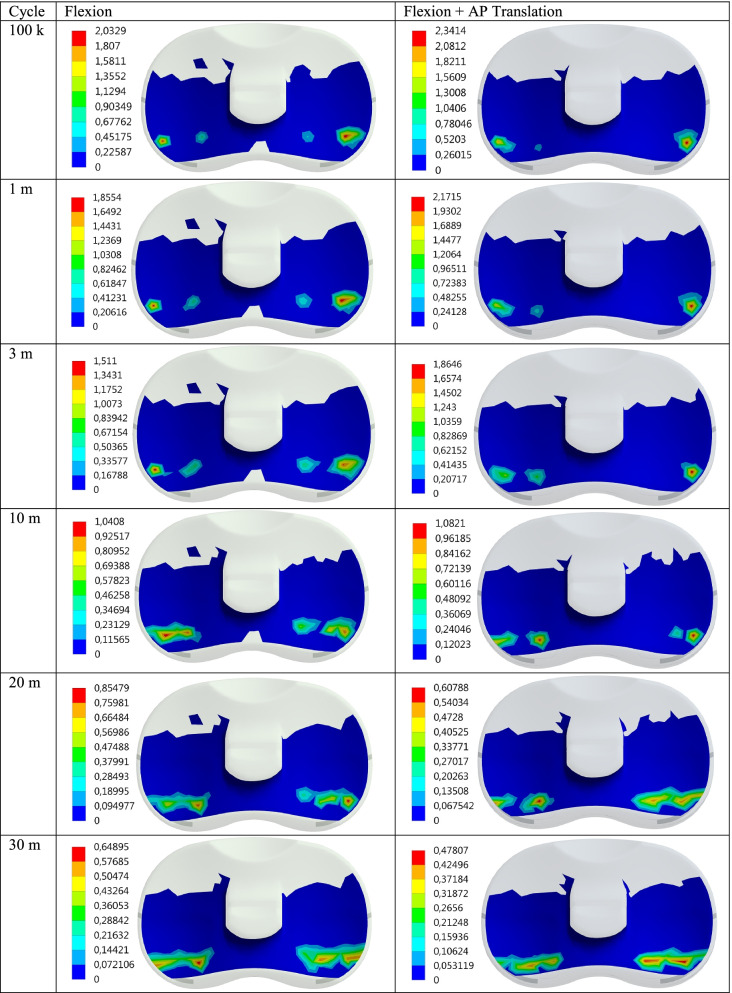
Max contact pressure distribution (MPa) on the PE insert surface for Flex and Flex+AP Translation wrt cycle

**Fig. 3:**
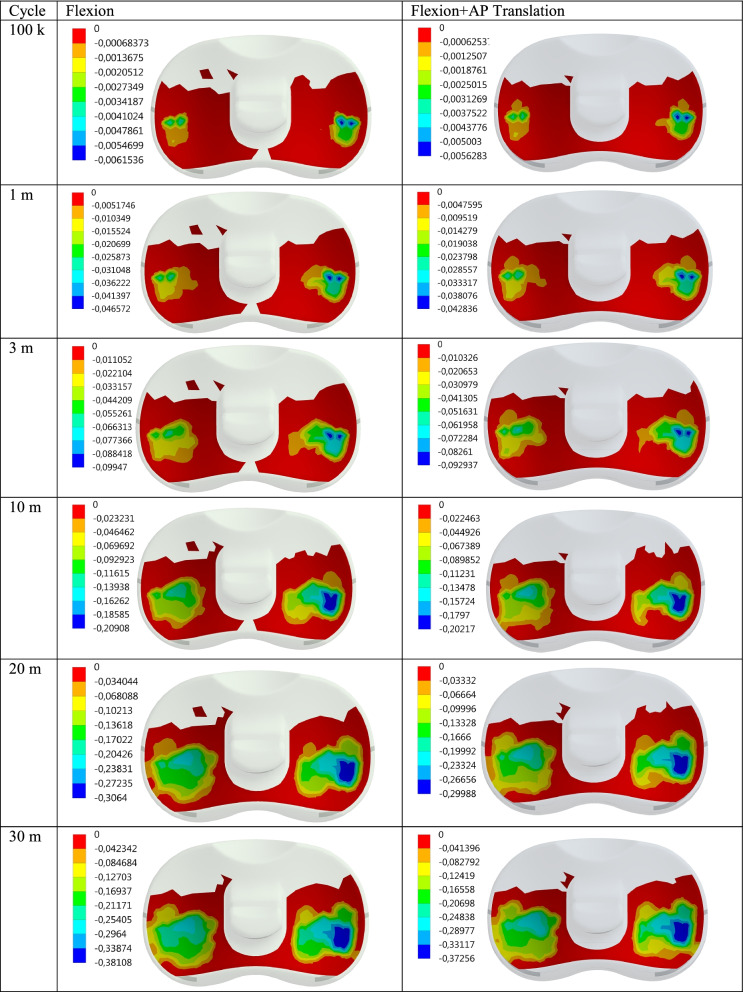
Wear distribution (mm) on the PE insert surface for Flex and Flex+AP Translation wrt cycle

Maximum contact pressure distributions are given for 100 thousand (100 k), 1, 3, 10, 20, and 30 million (30 m) cycles, respectively in Fig. 2. In addition, the contact pressure distributions show the effect of the AP Translation effect on the PE insert during flexion are also given in Fig. 3. Maximum contact pressure values obtained for Flexion and Flexion+AP Translation are given in Table 1 for better interpretation.

**Table 1:** Max contact pressure and wear data for Flexion and Flexion+AP Translation wrt cycle

	Flexion		Flexion+AP Translation	
Cycle	Press (MPa)	Wear (mm)	Press (MPa)	Wear (mm)
100 k	2.0329	0.0061536	2.3414	0.0056283
1 m	1.8554	0.046572	2.1715	0.042836
3 m	1.511	0.09947	1.8646	0.092937
10 m	1.0408	0.20908	1.0821	0.20217
20 m	0.85479	0.3064	0.60788	0.29988
30 m	0.64895	0.38108	0.47807	0.37256

It is seen that the contact pressure on the PE insert decreases as the cycle increases for both Flexion and Flexion+AP Translation. Maximum contact pressure values for Flexion and Flexion+AP Translation for 100 k cycles were obtained as 2.03 and 2.34 MPa, respectively. Here, while the maximum contact pressure at maximum flexion was 2.03 MPa for 100 k cycles, when the cycle was increased 10 times, that is, for 1 m cycles, it decreases to 1.86 MPa and the drop rate becomes 1.228. While the increase in the maximum contact pressure caused by the AP Translation, together with the cycles, reaches 1.23 in 3 m cycles; when the cycle reaches 10 m, the ratio decreases and it is seen that the contact pressure values for 20-30 m cycles are less than the values for flexion only.

**Fig. 4:**
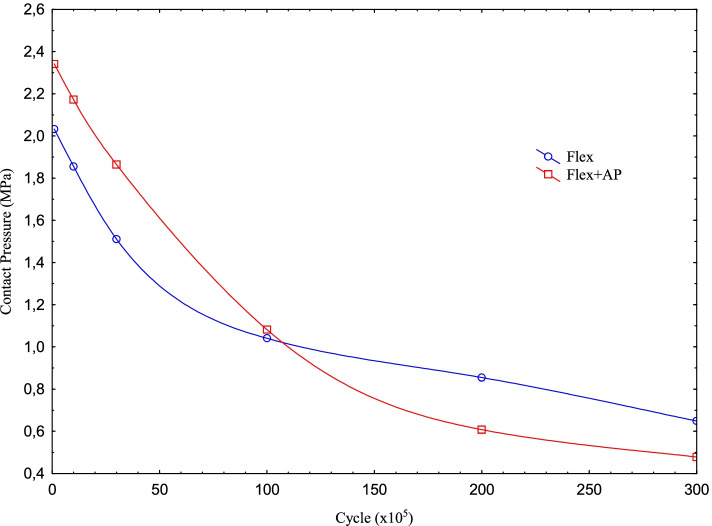
Max contact pressure distribution for Flexion and Flexion+AP Translation with the cycle at the contact interface

**Fig. 5:**
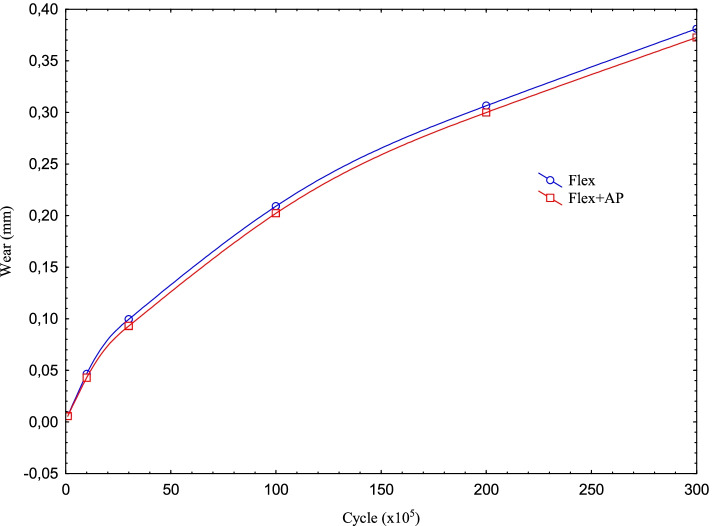
Wear distribution for Flexion and Flexion+AP Translation with the cycle at the contact interface

When the cycle is 1 m, i.e. 100 times the initial rate, the amount of pressure drops by almost half. In the case of flexion only, while the maximum contact pressure is 2.03 MPa for 100 k cycles, the pressure drops to 0.65 MPa at 30 m. As expected, this can be explained by the increase in wear and the corresponding increase in the contact area as the cycle increases. We can verify this from the increase in the wear corresponding to the cycle (Figs. 4 and 5). While the wear in “only flexion” is 0.006 mm for 100 k, this value increases to 0.38 mm at 30 m. For Flexion+AP Translation, it increases from 0.0056 mm to 0.37 mm.

It is clear that as the cycle increases, the wear obtained for both cases increases (Fig. 4). In addition, the wear on PE insert in the Flexion only for 100 k, 1 million and 3 million cycles are higher than the Flexion+AP Translation and while the initial wear is 1.09, with the increase in the cycles, this rate decreases to 1.02 at 30 m.

For Flexion+AP Translation, the pressure value drops from 2.34 MPa to 0.48 MPa. For Flexion+AP, the maximum contact pressure on the PE insert decreases as the cycle increases, just like in Flexion only. It is seen that AP Translation together with flexion increases the contact pressure by approximately 1.15. This shows us that the AP Translation is much more effective as the cycle increases tenfold from 10 million onwards. In other words, the loadings acting on the PE insert cannot create sufficient pressure due to the AP Translation effect at low speeds and have an effect to reduce the wear, while the effect increases with the wear as the cycle increases, and the AP Translation now contributes to the wear at high speeds.

### Tibial slope

**Fig. 6:**
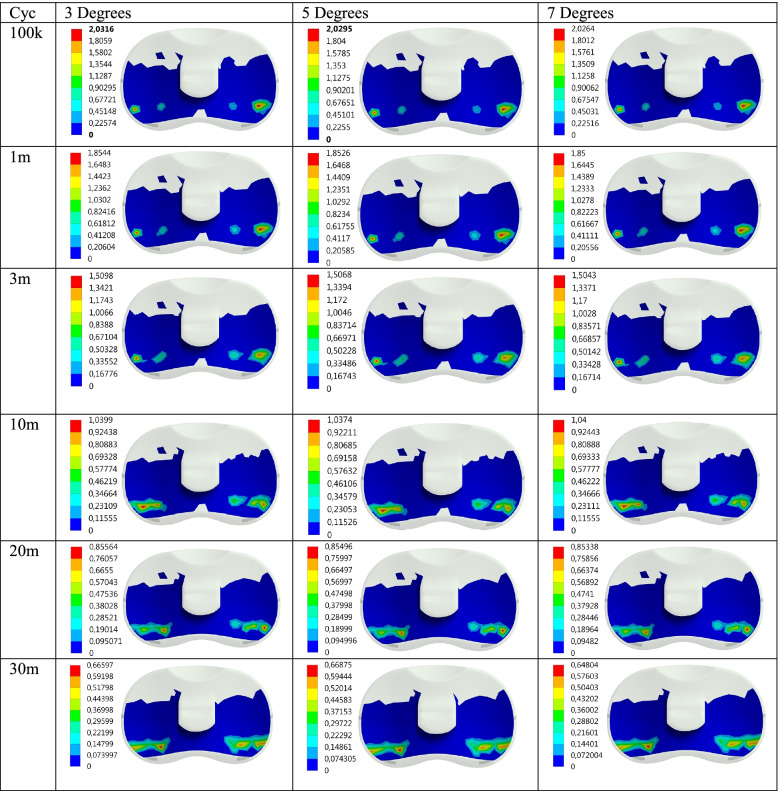
Max contact pressure distribution (MPa) on the PE insert surface for Posterior Tibial Slope degree wrt cycle

**Fig. 7:**
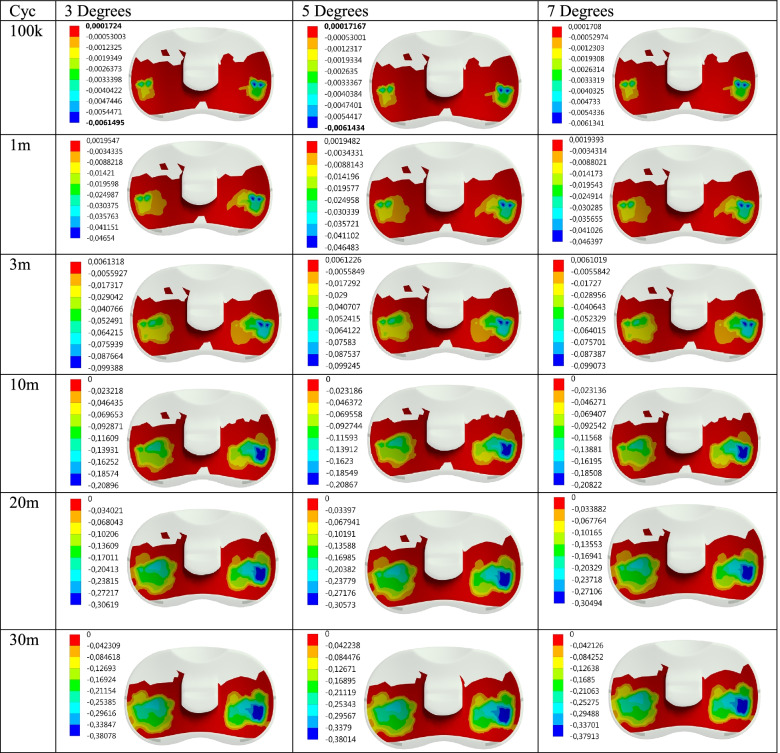
Wear distribution (mm) on the PE insert surface for Posterior Tibial Slope degree wrt cycle

Another concern of this study was to examine the effect of Posterior Tibial slope angle change on the contact surface of the PE Insert in the knee implant, on the maximum pressure distribution and wear, under much more realistic loading obtained by using a cadaver. The pressure and wear distributions obtained after the analyses are given in Figs. 6 and 7 and also in Table 2.

**Table 2:** Max contact pressure and wear data for Posterior Tibial Slope degree variation wrt cycle

	(Tibial Slope) Flexion					
	3 Degrees		5 Degrees		7 Degrees	
Cycle	Press (MPa)	Wear (mm)	Press (MPa)	Wear (mm)	Press (MPa)	Wear (mm)
100 k	2.0316	0.0061495	2.0295	0.0061434	2.0264	0.0061341
1 m	1.8544	0.04654	1.8526	0.046483	1.85	0.046397
3 m	1.5098	0.099388	1.5068	0.099245	1.5043	0.099073
10 m	1.0399	0.20896	1.0374	0.20867	1.04	0.20822
20 m	0.85564	0.30619	0.85496	0.30573	0.85338	0.30494
30 m	0.66597	0.38078	0.66875	0.38014	0.64804	0.37913

**Fig. 8:**
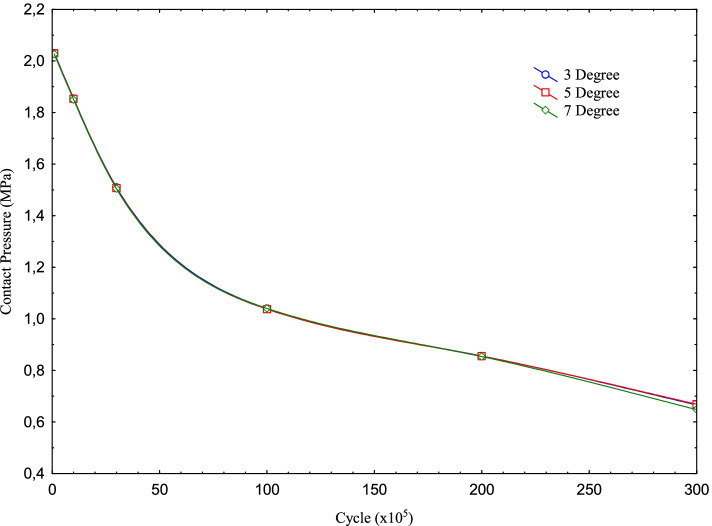
Max contact pressure distribution for Posterior Tibial Slope degree variation with the cycle at the contact interface

**Fig. 9:**
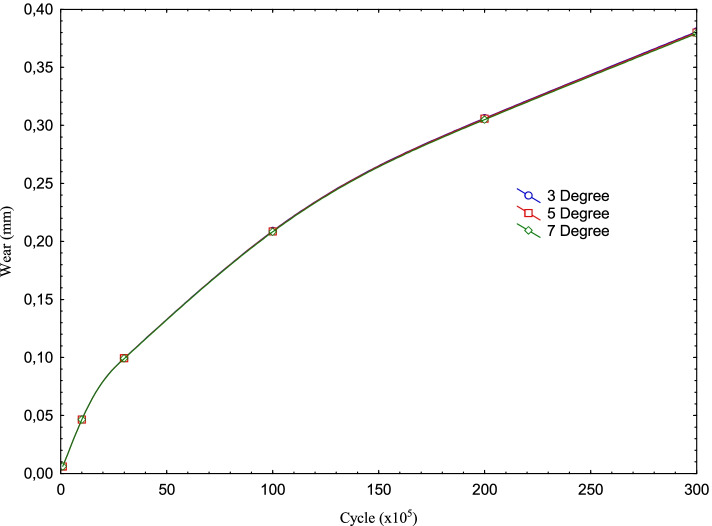
Wear distribution for Posterior Tibial Slope degree variation with the cycle at the contact interface

As seen in Table 2, in this study, analyzes were performed for knee implants with the posterior inclination of 3, 5, and 7 degrees in the above-mentioned loading condition for 6 different cycles under dynamic flexion varying between 0 and 90 degrees in increments of 5 degrees each. As seen in Fig. 8, the maximum contact pressure distributions on the PE insert obtained as a result of the analyses performed for the knee implant with a tibial slope of 3, 5 and 7 degrees decrease as the cycle increases. On the other hand, the wear increases as seen in Fig. 9.

It is seen that as the posterior tibial slope angle increases, the maximum contact pressure values slightly decrease for the same cycle. In other words, while the contact pressure is 2.0316 MPa for 100 k cycles and 3 degrees; for 5 degrees, this value is 2.0295 MPa, for 7 degrees it is 2.0264 MPa. Although this situation becomes somewhat irregular as the cycle increase, it shows that the trend is not deteriorated in general. On the other hand, it is seen that while the wear increases as the cycle increases for each tibial slope, it decreases as the tibial slope increases for each cycle. In other words, the pressure values obtained for 100 k are 2.0316 MPa, 2.0295 MPa, and 2.0264 MPa for the tibial slope of 3, 5, and 7 degrees; the wear decreases in the same angle order. As seen in Fig. 9, while the upward trend in the amount of wear was close until 3 m cycles, the amount of increase went up after this period.

It is seen that the contact pressure decreases, though with small differences, as the Tibial slope angle increases for 100 k, 1 m, and 3 m cycles. For 5 degrees Tibial slope, the contact pressure at 10 m cycles is less than other angles while it is the same for the other angles. In the analyses made for 20 m, it is seen that the pressure tends to decrease again with the increase in angle. In the pressure values obtained for 30 m, a result similar to the result in 10 m cycles was obtained. That is, the pressure value obtained for 5 degrees was higher than the other angles, while the value obtained for 7 degrees was the lowest. On the other hand, the wear increases as the cycle increases for each angle. While this upward trend is high at the beginning, it decreases as the cycle increases. However, as the Tibial slope angle increases with the cycles, it is seen that the wear decreases slightly.

## Discussion

As expected, the maximum contact pressure value for both analyzes decreases as the number of rotations increases. However, for both types of analysis, this decrease in pressure was very close up to 3 m cycles, while the decrease became more stable in the absence of AP Translation at 10 m. The slope in the AP Translation showed itself up to 20 m cycles. After 20 m cycles, the decreasing slopes continued close to each other. The fact that the pressure continued to decrease at almost the same slope as the cycle after 3 m cycles could be explained as the effect of AP Translation. The stabilization of the maximum pressure due to the effect of AP Translation took effect after 20 m cycles.

For both types of analysis, the wear, as was the case in maximum pressure distribution, had a similar trend up to 3 m cycles and the values were close to each other. After this period, the wear increase trends decreased for both analyzes. The decreasing trends of the analyzes remained almost the same as the number of cycles increased. On the other hand, there is some difference in the wear. Although the AP Translation increases the maximum contact pressure at low speeds and decreases it at high speeds, in this study, the wear is reduced by small amounts after 1 m cycles**. As** given in Wünschel et al. [30], the AP Translation data changes direction depending on the flexion angle. This shift of direction changes the nature of the problem according to the loading condition where only the Femur Component rotates according to flexion.

Now, while the femoral component rotates on the insert, the insert also makes a sliding motion in the AP direction in contact with the femoral component. This causes a difference in both the maximum contact pressure distribution and the wear distribution compared to the other model. It has been stated in previous studies that the rotational movement of the femur component on the insert due to flexion with respect to the femoral axis includes both rolling and slipping, whereas slipping is dominant in this movement [32, 33]. In addition, the AP Translation causes additional sliding motion. This additional movement, especially after 3 m cycles, prevents the transfer of the loading causing wear to the contact area, resulting in the amount of wear to be slightly lower. It causes the maximum contact pressure to occur in the left region. However, the maximum wear continues to take place in the right region.

Although the AP Translation initially increased the pressure a little and decreased the wear, it had an effect on reducing the pressure and still reducing the amount of wear in 10 m and higher cycles. This can be explained by the fact that AP Translation up to 10 m effectively increases the slip, thereby reducing the loading effect on the insert. In other words, it causes the loading to act on a smaller area and the pressure to be higher. However, at high cycles, this effect diminishes and the contact area increases while the pressure decreases. On the other hand, the lower wear can be attributed to the larger wear area.

As given in the Results section, as the cycle increases for each Posterior Tibial slope angle, the maximum contact pressure on the PE insert decreases and accordingly the wear increases. However, it is seen that the wear decreases as the posterior tibial slope angle increases with increasing cycles. This is because as the angle increases, one of the 50 Newtons force on the connection interface is still perpendicular to the contact interface while the other is divided into two forces that start to act parallel to the contact interface and the one perpendicular to the contact interface decreases and the parallel one increases. In other words, while the perpendicular force is 49.93 N for an angle of 3 degrees, it decreases to 49.81 for 5 degrees and 49.63 N at 7 degrees. On the other hand, the force parallel to the contact interface is 2.62, 4.36, and 6.09 N for 3, 5, and 7 degrees, respectively.

The loading used in this study shows that as the cycle increases for AP Translation and AP Tibial slope angle, the wear area on the PE insert enlarges and approaches the Posterior. While this situation is not very evident in relatively low cycles such as 100 k and 1 m, it becomes evident in cycles such as 20 m and 30 m. This can be because the knee implant used in this study is a model called cruciate sacrificing total knee replacement. In this model, as the femoral component rotates with flexion, after a certain angle, it touches the projection designed to function as the posterior cruciate ligament on the PE insert and erodes this region more.

The slight decrease in the wear with posterior tibial slope angle is due to the fact that the vertical loading force, which is perpendicular to the PE insert-Femur Component interface at 0 degree and taken as 50 N in this study, splits into two components as perpendicular and parallel to the contact interface as the mounting angle changes, and that the parallel force increases and the perpendicular force decreases as the angle increases. In addition, it is seen as an advantage to use a bigger angle in the knee implant since the wear is reduced as the mounting angle, i.e. the Posterior Slope angle increases. On the other hand, although using a bigger angle is seen as an advantage, as the angle increases, the force acting parallel to the contact interface will increase, forcing the interface to slide even in the static state. For this reason, it will be useful to carry out additional experimental and numerical studies to determine the static and dynamic nature of the Posterior Slope angle change together with the direction of the forces coming to the interface, flexion, AP Translation, and other factors.

This study demonstrated that the wear distributions (based on cycle) drawn according to the data obtained as a result of the simulations were qualitatively similar to the experimental studies conducted by Kawanabe et al. [23]. These findings also revealed that the correct result was obtained depending on the selected wear model. In addition, although the loading inputs were different, the wear amount distributions were qualitatively similar to the numerical study of Zhang et al. [20] up to 15 k cycles. On the other hand, unlike previous studies, the wear was found to continue to increase as the number of cycles increased, although the slope decreased. It was found that the wear zone on the PE insert was obtained as a result of this study and the change with the cycle of this zone were in parallel with the “change of wear zones according to the number of rotations on the insert” obtained by Zhang et al. [20] who used a wear model from the literature.

## Conclusions

This study was limited in that it does not take into account tibial rotation and indicated that AP Translation, which changes direction during flexion, had a significant effect on both contact pressure and wear. Unlike previous similar studies, it was seen that the amount of wear continues to increase as the cycle increases. This situation strengthens the argument that loading and AP Translation values that change with flexion shape the wear effects on PE Insert. It is seen that the posterior tibial slope angle - which occupies an important place in the amount of wear on the insert and the load coming to the contact interface in the knee implant mounting - increases with the cycles, and the wear slightly decreases.

## Data Availability

All data generated or analyzed during this study are included in this manuscript.
